# Clinical Course and Long-Term Outcome of Hantavirus-Associated Nephropathia Epidemica, Germany

**DOI:** 10.3201/eid2101.140861

**Published:** 2015-01

**Authors:** Joerg Latus, Matthias Schwab, Evelina Tacconelli, Friedrich-Michael Pieper, Daniel Wegener, Juergen Dippon, Simon Müller, David Zakim, Stephan Segerer, Daniel Kitterer, Martin Priwitzer, Barbara Mezger, Birgit Walter-Frank, Angela Corea, Albrecht Wiedenmann, Stefan Brockmann, Christoph Pöhlmann, M. Dominik Alscher, Niko Braun

**Affiliations:** Robert-Bosch-Hospital, Stuttgart, Germany (J. Latus, F.-M. Pieper, D. Wegener, D. Zakim, D. Kitterer, C. Pöhlmann, M.D. Alscher, N. Braun);; Dr. Margarete Fischer-Bosch-Institute of Clinical Pharmacology, Stuttgart (M. Schwab);; University Hospital Tübingen, Germany (M. Schwab, E. Tacconelli);; University of Stuttgart, Stuttgart (J. Dippon, S. Müller);; University Hospital Zurich, Zurich, Switzerland (S. Segerer);; Local Health Authority, Stuttgart (M. Priwitzer, B. Mezger);; Local Health Authority, Böblingen, Germany (B. Walter-Frank);; Local Health Authority, Esslingen, Germany (A. Corea, A. Wiedenmann);; Local Health Authority, Reutlingen, Germany (S. Brockmann)

**Keywords:** Hantavirus, PUUV, Puumala virus, nephropathia epidemica, epidemic nephropathy, long-term outcome, hematuria, hypertension, proteinuria, viruses, acute kidney injury, Germany

## Abstract

Human infection with Puumala virus (PUUV), the most common hantavirus in Central Europe, causes nephropathia epidemica (NE), a disease characterized by acute kidney injury and thrombocytopenia. To determine the clinical phenotype of hantavirus-infected patients and their long-term outcome and humoral immunity to PUUV, we conducted a cross-sectional prospective survey of 456 patients in Germany with clinically and serologically confirmed hantavirus-associated NE during 2001–2012. Prominent clinical findings during acute NE were fever and back/limb pain, and 88% of the patients had acute kidney injury. At follow-up (7–35 mo), all patients had detectable hantavirus-specific IgG; 8.5% had persistent IgM; 25% had hematuria; 23% had hypertension (new diagnosis for 67%); and 7% had proteinuria. NE-associated hypertension and proteinuria do not appear to have long-term consequences, but NE-associated hematuria may. All patients in this study had hantavirus-specific IgG up to years after the infection.

## Abstract 2

The consequences of associated hematuria may be long-lasting, and hantavirus IgG is detectable years after acute infection.

Hantaviruses, enveloped RNA viruses of the family *Bunyaviridae*, are transmitted to humans by rodents, the natural reservoir of these viruses (*1*). In North America, hantavirus infection can lead to hantavirus cardiopulmonary syndrome and to case–fatality rates of up to 35% (*2*,*3*), and in Asia and Europe, infection can lead to hemorrhagic fever with renal syndrome (HFRS) (*4*).

In Germany, the incidence of HFRS increased from 0.09 cases/100,000 persons in 2001 to 2.47 cases/100,000 persons in 2010 (*5*). During October 2011–April 2012, a total of 852 HFRS cases were reported in Germany, of which 580 (68%) originated in the southern federal state of Baden-Wurttemberg (*6*). Puumala virus (PUUV), by far the most frequent cause of hantavirus disease in Germany (*7*), causes a milder form of HFRS (*8*) called nephropathia epidemica (NE). Hantavirus infections are one of the 5 most common notifiable viral diseases in Germany, along with norovirus infections, hepatitis C, influenza, and rotavirus infections (*7*).

NE is characterized by acute kidney injury associated with thrombocytopenia and, frequently, with proteinuria (*9*). Severe and often prolonged gastrointestinal symptoms and severe back and abdominal pain also occur (*10*). The severity of infection with PUUV varies from subclinical disease to severe acute kidney injury, including a fatal outcome (*11*,*12*). Renal replacement therapy is required in ≈5% of hospitalized patients with acute NE (*8*,*10*,*13*,*14*), although some studies report rates of up to 25% (*15*,*16*).

Despite the high and increasing incidence of hantavirus infection, long-term follow-up data have not been reported for a large, representative cohort of patients. Moreover, it remains unclear whether NE has long-term consequences, such as hypertension, proteinuria (*17*), and alteration of kidney function (*18*,*19*). Two previous studies that followed up 36 Finnish patients 5 and 10 years after they experienced PUUV-associated NE, reported that the patients had increased urinary protein excretion, glomerular hyperfiltration, and elevated blood pressure at a 5-year but not a 10-year follow-up (*18*,*19*). Other reports support an association between previous hantavirus infection and subsequent hypertension (*20*–*23*).

Data on humoral immunity years after PUUV infection are not available for a representative cohort of German patients. Thus, we conducted this study in such a cohort to describe the detailed clinical phenotype of patients with clinical manifestations of PUUV infection and their long-term outcomes and humoral immunity to PUUV.

## Patients and Methods

### Patients

Since 2001, Germany’s Protection against Infection Act (http://www.rki.de/EN/Content/Prevention/Inf_Dis_Surveillance/inf_dis_down.pdf?__blob=publicationFile, section 7) has required that confirmed cases of hantavirus infection be reported (on a named-patient basis) to local health authorities. In turn, the health authorities report HFRS cases to the Robert Koch Institute in Berlin, Germany, the central federal institution responsible for disease control and prevention.

During 2001–2012, a total of 7,476 serologically and clinically confirmed hantavirus cases were reported to the Robert Koch Institute (SurvStat, https://www.survstat.rki.de/) (*24*). In cooperation with 4 local health authorities in southern Germany (Stuttgart, Boeblingen/Sindelfingen, Esslingen, Reutlingen), we identified 1,570 (21%) of 7,476 persons who had received a clinically and serologically confirmed diagnosis of hantavirus-associated NE during 2001–2012. During September 2012–April 2013, we contacted these persons by mail, asking them to come for a follow-up examination at the outpatient clinic of Robert-Bosch-Hospital (Stuttgart). All patients gave written consent before participating in the study. The study was approved by the Ethics Committee of the Ethics Commission of the State Chamber of Medicine in Baden-Wuerttemberg (Stuttgart) (approval no. F-2012–046). Studies were conducted in concordance with the Declaration of Helsinki.

### Data Acquisition

#### Acute Course of NE

Data on clinical and laboratory findings at the time of diagnosis and during the acute course of the disease were obtained from medical reports and files for each patient. Details of data acquisition are shown in the Technical Appendix.

#### Follow-up

All patients (i.e., those treated as inpatients and those treated as outpatients) were included in the follow-up. Patients participated in a follow-up session once at the Robert-Bosch-Hospital outpatient clinic. Detailed past and current medical histories were obtained by using standardized case report forms, and a physical examination was conducted. The case report form, which was designed after a systematic review of the literature, included 56 questions divided into 3 sections: demographic data, time of diagnosis and acute course of the disease, and follow-up. Blood pressure for all patients was measured as recommended by the American Heart Association (*25*); results were classified as normotensive, prehypertension, hypertension stage 1, or hypertension stage 2, according to the classification of blood pressure for adults as described in the Seventh Report of the Joint National Committee on Prevention, Detection, Evaluation, and Treatment of High Blood Pressure (*26*) (Technical Appendix).

At the follow-up appointment, standard procedures were used by the Robert-Bosch-Hospital laboratory to determine complete blood counts and levels of plasma C-reactive protein, serum creatinine, urea, and second-morning urine and to perform liver function tests and blood gas analysis. Proteinuria was defined as microalbuminuria (>30 mg albumin/24 h) in urine. In addition, serum samples from all patients were analyzed for PUUV-specific IgM and IgG by using a strip-immunoassay (*recom*Line Bunyavirus IgG/IgM, Mikrogen, Neuried, Germany).

### Statistical Analysis

Laboratory findings were tabulated as medians and interquartile ranges (IQRs). Clinical symptoms were enumerated by percentage of affected patients.

## Results

### Clinical Findings and Course of Acute NE

During September 2012–April 2013, we contacted (by mail) 1,570 persons in Baden-Wurttemberg State, Germany, for possible inclusion in this study; all of these persons had received a diagnosis of serologically confirmed NE during 2001–2012. Of the 1,570 persons, 456 (29%) were included in the study, representing 6.1% of ever-reported cases of hantavirus infection in Germany. Reasons for exclusion included failure to respond to the request (1,104 patients), being <18 years of age at the time of diagnosis (3 patients), and missed follow-up appointment (10 patients) (Technical Appendix).

Median age at diagnosis was 48 years (IQR 40–59). Of the 456 patients, 290 (64%) were male and 166 (36%) were female; the higher number of cases in men corresponds to the characteristics of the overall reported cases in Germany during 2001–2013 (Survstat, https://www.survstat.rki.de/). Of the 456 patients, 335 had received inpatient treatment, and 121 had received outpatient treatment by general practitioners or nephrologists. Time from onset of symptoms to admission to hospital or first contact with a general practitioner/nephrologist was 5 days (range 3–7). Prominent clinical findings were fever (90%), back pain (67%), limb pain (71%), and nausea and/or vomiting (47%). Baseline characteristics of the study population during the acute course of the disease are summarized in Table 1. At hospital admission/first visit to the general practitioner/nephrologist, 70% of the patients exhibited acute kidney injury, as determined by RIFLE (risk of renal dysfunction) criteria (*27*). Thrombocytopenia was present in 61% of patients at hospital admission/first visit with a general practitioner/nephrologist. Severe thrombocytopenia (platelet count <60 × 10^9^/L) was found in 49 patients (11%), none of whom required platelet transfusion. Mild hemorrhagic symptoms, mostly epistaxis, occurred in 7% of the patients; no patients had severe bleeding complications. Lymphocytopenia was found in 55% of patients, monocytosis was observed in 59% at hospital admission/first visit to the general practitioner/nephrologist and C-reactive protein levels were substantially elevated in 96% of patients. Baseline characteristics are summarized in Table 1 and Table 2.

**Table 1:** Baseline characteristics for 456 persons during acute hantavirus infection, Germany, 2001–2012

Variable	Median value (range) or no. or % patients positive for variable
Sex, no.	
M	290
F	166
Age at diagnosis	48 y (40–59)
Patient status, no.	
Inpatient	335
Outpatient	121
Duration of hospital stay	7 d (5–9)
Duration of illness at hospital admission or first visit to general practitioner/nephrologist	5 d (3–7)
Duration of fever before hospital admission	4 d (2–6)
Duration of disease	4 wk (2–8)
Assumed exposure to virus, %	
Occupational	7
During leisure activities	93
Symptoms, %	
Pain	
Abdominal	33
Back	67
Limbs	71
Headache	68
Visual disorders	22
Diarrhea	20
Nausea/vomiting	47
Dyspnea	2
Loss of appetite	4
Fatigue	7
Shivering	2
Clinical signs	
Fever, %	90
Duration of fever	5 d (3–7)
Hemorrhage, %	7
Blood pressure	
Systolic,	130 mm Hg (120–146)
Diastolic	80 mm Hg (71–90)
Heart rate	76 beats/min (64–86)
Hypertension, %	15
Hypotension, %	10
Shock, %	1
Chest radiograph, %	47
Pathologic findings on radiograph, %	23
Abdominal ultrasound, %	65
Splenomegaly, %	14
Hepatomegaly, %	4
Acute kidney injury, %*	86

*According to RIFLE (risk of renal dysfunction) criteria (*27*).

**Table 2:** Laboratory results for serum samples obtained from 456 persons experiencing acute hantavirus-associated nephropathia epidemica, Germany, 2001–2012*

Laboratory test	Median value (range)	Reference value
Creatinine, serum, maximum	2.7 mg/dL (1.6–4.8)	0.5–1.4 mg/dL
Platelet count		>150 × 10^9^/L
At admission or first visit with a general practitioner/nephrologist	122 (81–197)	
Minimum	113 (75–189)	
At discharge§	281 (226–353)	
Hematocrit		0.37–0.47
At admission or first visit with a general practitioner/nephrologist	0.42 (0.39–0.47)	
At discharge	0.40 (0.36–0.43)	
C-reactive protein		0.1–0.4 mg/dL
At admission or first visit with a general practitioner/nephrologist	4 mg/dL (2.4–7.1)	
Maximum	4.5 mg/dL (2.8–8.2)	
At discharge§	0.9 mg/dL (0.4–1.7)	
Liver enzyme†		
Aspartate aminotransferase		<50 U/L
At admission or first visit with a general practitioner/nephrologist	36 U/L (27–55)	
At discharge§	51 U/L (30–71)	
Alanine aminotransferase		<50 U/L
At admission or first visit with a general practitioner/nephrologist	35 U/L (24–57)	
At discharge§	64 U/L (35–101)	
Lactate dehydrogenase‡	273 U/L (240–323)	<250 U/L

*All patients were adults who had received a diagnosis of serologically and clinically confirmed hantavirus infection. †35% of patients had elevated levels of liver enzymes at admission or first visit with a general practitioner/nephrologist. ‡66% of patients had elevated levels of lactate dehydrogenase at admission or first visit with a general practitioner/nephrologist. §335 patients who received in-patient treatment.

### Follow-up

Baseline characteristics of the study population at the follow-up examination are summarized in Table 3. The median time between diagnosis with acute disease and follow-up was 17 months (IQR 7–35). Fifty per cent of patients had NE >2 years before the follow-up visit, and 20% had NE >5 years before the follow-up visit. All but 2 patients had serum creatinine levels within the reference range at follow-up. One of these 2 patients had hypertension and coronary disease before onset of NE; the second patient received a diagnosis of glomerulonephritis 3 months after the onset of NE. Thrombocytopenia was still present in 4% of the patients at follow-up. Proteinuria was present in 34 patients (7%). None of the patients had symptoms of urinary tract infection at follow-up. Urinanalysis showed a high proportion of patients with hematuria (26%). We compared baseline characteristics (e.g., laboratory values, severity of acute kidney injury, symptoms during acute course of the disease) and findings (e.g., kidney function, blood pressure, and protein levels in urine) from the acute course of the disease for patients with and those without hematuria at follow-up: no statistically significant differences were observed between the groups (data not shown).

**Table 3:** Baseline characteristics for 456 participants in a follow-up study to determine the clinical course and long-term outcome of hantavirus-associated nephropathia epidemica, Germany, 2001–2012*

Variable	Median value (range) or % patients positive for variable	Reference value
Laboratory test		
Creatinine, serum	0.9 mg/dL (0.8–1.0)	0.5–1.4 mg/dL
Blood cell counts		
Leukocytes	6.7 × 10^9^/L (5.7–7.9)	4.0–11.3 × 10^9^/L
Platelets	231 × 10^9^/L (201–268)	>150 × 10^9^/L
Hemoglobin	14.9 g/dL (14.1–15.6)	13–18 g/dL
Hematocrit	0.43 (0.42–0.46)	0.37–0.47
Uric acid	5.2 mg/dL (4.3–6.0)	4.8–5.6 mg/dL
Liver enzymes		
Aspartate aminotransferase	21 U/L (17–25)	<50 U/L
Alanine aminotransferase	21 U/L (15–30)	<50 U/L
Lactate dehydrogenase	158 U/L (142–180)	<250 U/L
C-reactive protein	0.1 mg/dL (0.1–0.2)	0.1–0.4 mg/dL
Urinalysis		
Proteinuria	7	–
Hematuria	26	–
Leukocyuria	15	–
Presence of bacteria	0	–
Hantavirus-specific antibodies		
IgM present	9	–
IgG present	100	–
Clinical test		
Blood pressure†		
Systolic	135 mm Hg (125–148)	–
Diastolic	83 mm Hg (76–90)	–
Hypertension at first follow-up, 203/456 participants	45	–
Stage 1 hypertension, 98/203 hypertensive patients	48	–
Stage 2 hypertension, 105/203 hypertensive patients	52	–
Hypertension at second follow-up up‡	23	–
Stage 1 hypertension	66	–
Stage 2 hypertension	34	–
Heart rate	70 beats/min (59–79)	–
Use antihypertensive drugs	43	–
Family history of hypertension	60	–
Smoke cigarettes/cigars	33	–
Concomitant condition		
Coronary heart disease	4	–
Peripheral arterial disease	1	–
Heart failure	2	–
Diabetes mellitus	3	–

*Study participants were adults who had received a diagnosis of serologically and clinically confirmed hantavirus infection during 2001–2012 and who later (7–35 mo) participated in a follow-up study. –, not applicable. †Hypertension stages 1 and 2 were defined according to the classification of blood pressure for adults (*26*). ‡Includes only those participants who had hypertension at the first follow-up visit. Retesting was done within 8 wk after first follow-up visit.

Blood pressure was measured twice for all patients in the outpatient clinic. In this setting, 203 (45%) of the 456 patients had hypertension stage 1 or 2, according to the classification of blood pressure for adults (*26*). All of these patients were contacted again within 8 weeks after the appointment in the outpatient clinic to reevaluate blood pressure measurements in the ambulatory setting. On the basis of these data, hypertension was present in 23% of all patients at follow-up. One third of these hypertensive patients had documented preexisting hypertension. In patients with preexisting hypertension, there was no change in blood pressure after NE, and antihypertensive therapy was not increased to achieve pre-NE blood pressure values.

Serum samples from all patients were analyzed for hantavirus-specific IgM and IgG at follow-up. All patients had detectable IgG, and 8.5% had persistent IgM.

## Discussion

The data from our study show that NE is responsible for severe acute kidney injury in a high proportion of patients with PUUV infection. Proteinuria and hematuria were frequently present during the acute course of NE. In contrast to findings in previously published small studies (*18*,*19*), our findings show that proteinuria and hypertension are not long-term sequelae of the disease. Of note, hematuria was present during the acute course of disease and at long-term follow-up (median 17 mo [IQR 7–35 mo]). Furthermore, our data show that neutralizing antibodies are still present up to years after infection, and no recurrent disease was reported. During the acute course of PUUV infection, the disease causes acute kidney injury in 86% of patients. In our study, only 3% of the patients required transient renal replacement therapy; this percentage is lower than that reported in previous smaller studies (*8*,*10*,*13*–*16*). Compared with previous studies, our study used well-defined, consistent classification criteria to describe the clinical course of patients with NE (*10*,*28*,*29*). Apart from acute kidney injury, NE is clinically characterized by thrombocytopenia and, frequently, proteinuria (*8*,*9*,*30*,*31*). Thrombocytopenia was present in 219 (61%) of the patients in our study, and severe thrombocytopenia (platelet count <60 × 10^9^/L) was found in 49 (11%) patients. Of interest, none of the patients required platelet transfusion, and only mild hemorrhagic symptoms, mostly epistaxis, occurred in a small percentage (7%) of all patients, and the hemorrhagic symptoms occurred independently of thrombocyte counts. Previous smaller studies reported higher rates of bleeding complications in patients with NE (*32*,*33*). At hospital admission, the thrombocyte nadir had been reached in 90% of the patients; this finding supports the hypothesis that thrombocytopenia occurs in early stages of the disease. Furthermore, visual disorders were present in 22% of our study population; the disorders, caused by a swelling of the lens, are often one of the first symptoms of NE (*34*). Therefore, NE should be considered in patients with acute onset of blurred vision and fever, especially in areas where the disease is endemic.

Reports on the long-term outcome of NE in large cohorts of patients are lacking. Hypertension has been discussed as a long-term consequence of NE (*17*), and 2 previous follow-up studies of 36 patients in Finland (5 and 10 years after infection) reported that patients exhibited increased urinary protein excretion, glomerular hyperfiltration, and elevated blood pressure at the 5- but not the 10-year follow-up (*18*,*19*). Some reports have suggested an association between previous hantavirus infection and subsequent hypertension (*20*–*23*). In our study population, 23% of patients had hypertension at follow-up. It is noteworthy, however, that these patients were older (45–63 years of age) than the patients without hypertension. Furthermore, the incidence of high blood pressure in patients in our study was not different than that in an age-matched cohort of patients without a history of NE (*35*). In contrast to findings in previous studies (*20*–*23*), we did not find that hypertension was a frequent sequela of NE in our study population. Persistent proteinuria following NE is a currently unresolved but frequently discussed issue. In our study, only 30 patients exhibited proteinuria at follow-up, and 19 (57%) of these patients had received a diagnosis of hypertension before the development of NE, suggesting that proteinuria does not seem to be long-term consequence of NE. At follow-up, a large proportion of patients had excess traces of blood in the urine (i.e., microscopic hematuria). Whether persistent hematuria after NE reflects lasting damage to glomerular cells, as a long-term consequence, remains uncertain because of the lack of renal biopsy specimens at long-term follow-up.

Representative data on humoral immune responses to PUUV in a large cohort of patients are lacking (*36*). Hantavirus-specific IgM and IgG were detected at initial diagnosis and during follow-up. All patients had hantavirus-specific IgG antibodies at follow-up, suggesting that neutralizing antibodies are still present up to years after infection.

Our study design had several limitations. First, only one third of NE cases occur with typical clinical signs and symptoms, which results in high underreporting, especially of younger patients with mild disease. Second, patients were contacted by mail and asked to attend the outpatient clinic for follow-up investigations, which may have led to a selection bias because more patients with a severe course of disease might have been included in the study. Third, we did a retrospective study of medical case reports for the patients during acute NE, associated with all known limitations. Previously published Finnish long-term outcome studies of patients with NE (*18*,*19*) investigated small cohorts of patients, but they provided accurate glomerular filtration rate measurements and 24-hour ambulatory blood pressure measurements and compared their results with those of a control group. Because of the large number of patients in our study and the wide geographic area in which they resided, we could not obtain 24-hour blood pressure monitoring; thus, we focused on blood pressure measurements obtained in the outpatient clinic at follow-up and on interviews with patients who had elevated blood pressure at that time. Therefore, establishment of a matched control group was not possible. The last possible limitation is that serum samples from PUUV-infected patients cross-react strongly with Sin Nombre virus and weakly with Hantaan virus, Seoul virus, and Dobrava virus (*36*,*37*). Although Dobrava-Belgrade virus and Tula virus circulate in rodent hosts in Germany and might cause an infection in humans (*38*–*40*), almost all hantavirus infections (especially in southern Germany) are caused by PUUV (*5*,*7*). This fact minimizes the risk for inclusion in our study of patients with HFRS caused by a hantavirus other than PUUV.

In summary, PUUV-associated NE is responsible for acute kidney injury in a high percentage of patients. Hypertension and proteinuria do not seem to be long-term consequences of NE, but hematuria may be, and patients should therefore be monitored after PUUV infection. The presence of hantavirus-specific IgG in patients after PUUV infection suggests that neutralizing antibodies can be present as long as years after infection.

Technical Appendix  Details of data acquisition from patients during acute course of nephropathia epidemica and flowchart showing design of hantavirus study, Germany, 2001–2012.

## References

[1] Schmaljohn CS, Dalrymple JM. Analysis of Hantaan virus RNA: evidence for a new genus of *Bunyaviridae.* Virology. 1983;131:482–91 . 10.1016/0042-6822(83)90514-7 6419460

[2] Krüger DH, Ulrich R, Lundkvist AA. Hantavirus infections and their prevention. Microbes Infect. 2001;3:1129–44. 10.1016/S1286-4579(01)01474-5 11709294

[3] Peters CJ, Simpson GL, Levy H. Spectrum of hantavirus infection: hemorrhagic fever with renal syndrome and hantavirus pulmonary syndrome. Annu Rev Med. 1999;50:531–45. 10.1146/annurev.med.50.1.531 10073292

[4] Watson DC, Sargianou M, Papa A, Chra P, Starakis I, Panos G. Epidemiology of hantavirus infections in humans: a comprehensive, global overview. Crit Rev Microbiol. 2014;40:261–72. 10.3109/1040841X.2013.783555 23607444

[5] Ettinger J, Hofmann J, Enders M, Tewald F, Oehme RM, Rosenfeld UM, Multiple synchronous outbreaks of Puumala virus, Germany, 2010. Emerg Infect Dis. 2012;18:1461–4 . 10.3201/eid1809.111447 22932394 PMC3437711

[6] Boone I, Wagner-Wiening C, Reil D, Jacob J, Rosenfeld UM, Ulrich RG, Rise in the number of notified human hantavirus infections since October 2011 in Baden-Wurttemberg, Germany. Euro Surveill. 2012;17:20180 . 22687824

[7] Krüger DH, Ulrich RG, Hofmann J. Hantaviruses as zoonotic pathogens in Germany. Dtsch Arztebl Int. 2013;110:461–7 . 23964302 10.3238/arztebl.2013.0461 PMC3722641

[8] Vapalahti O, Mustonen J, Lundkvist A, Henttonen H, Plyusnin A, Vaheri A. Hantavirus infections in Europe. Lancet Infect Dis. 2003;3:653–61. 10.1016/S1473-3099(03)00774-6 14522264

[9] Krautkrämer E, Zeier M, Plyusnin A. Hantavirus infection: an emerging infectious disease causing acute renal failure. Kidney Int. 2013;83:23–7. 10.1038/ki.2012.360 23151954

[10] Braun N, Haap M, Overkamp D, Kimmel M, Alscher MD, Lehnert H, Characterization and outcome following Puumala virus infection: a retrospective analysis of 75 cases. Nephrol Dial Transplant. 2010;25:2997–3003. 10.1093/ndt/gfq118 20223893

[11] Hjertqvist M, Klein SL, Ahlm C, Klingstrom J. Mortality rate patterns for hemorrhagic fever with renal syndrome caused by Puumala virus. Emerg Infect Dis. 2010;16:1584–6. 10.3201/eid1610.100242 20875284 PMC3294390

[12] Makary P, Kanerva M, Ollgren J, Virtanen MJ, Vapalahti O, Lyytikainen O. Disease burden of Puumala virus infections, 1995–2008. Epidemiol Infect. 2010;138:1484–92. 10.1017/S0950268810000087 20109263

[13] Mustonen J, Brummer-Korvenkontio M, Hedman K, Pasternack A, Pietila K, Vaheri A. Nephropathia epidemica in Finland: a retrospective study of 126 cases. Scand J Infect Dis. 1994;26:7–13. 10.3109/00365549409008583 7910705

[14] Settergren B, Juto P, Trollfors B, Wadell G, Norrby SR. Hemorrhagic complications and other clinical findings in nephropathia epidemica in Sweden: a study of 355 serologically verified cases. J Infect Dis. 1988;157:380–2. 10.1093/infdis/157.2.380 2891778

[15] Rasche FM, Uhel B, Kruger DH, Karges W, Czock D, Hampl W, Thrombocytopenia and acute renal failure in Puumala hantavirus infections. Emerg Infect Dis. 2004;10:1420–5 . 10.3201/eid1008.031069 15496243 PMC3320406

[16] Ala-Houhala I, Koskinen M, Ahola T, Harmoinen A, Kouri T, Laurila K, Increased glomerular permeability in patients with nephropathia epidemica caused by Puumala hantavirus. Nephrol Dial Transplant. 2002;17:246–52. 10.1093/ndt/17.2.246 11812874

[17] Niklasson B, Hellsten G, LeDuc J. Hemorrhagic fever with renal syndrome: a study of sequelae following nephropathia epidemica. Arch Virol. 1994;137:241–7. 10.1007/BF01309472 7944947

[18] Mäkelä S, Ala-Houhala I, Mustonen J, Koivisto AM, Kouri T, Turjanmaa V, Renal function and blood pressure five years after Puumala virus–induced nephropathy. Kidney Int. 2000;58:1711–8. 10.1046/j.1523-1755.2000.00332.x 11012905

[19] Miettinen MH, Mäkelä SM, Ala-Houhala IO, Huhtala HS, Koobi T, Vaheri AI, Ten-year prognosis of Puumala hantavirus–induced acute interstitial nephritis. Kidney Int. 2006;69:2043–8. 10.1038/sj.ki.5000334 16641933

[20] Lähdevirta J. Nephropathia epidemica in Finland. A clinical histological and epidemiological study. Ann Clin Res. 1971;3:1–54 . 5149038

[21] Lähdevirta J, Collan Y, Jokinen EJ, Hiltunen R. Renal sequelae to nephropathia epidemica. Acta Pathol Microbiol Scand [A]. 1978;86:265–71 . 31055 10.1111/j.1699-0463.1978.tb02042.x

[22] Rubini ME, Jablon S, Mc DM. Renal residuals of acute epidemic hemorrhagic fever. Arch Intern Med. 1960;106:378–87. 10.1001/archinte.1960.03820030066011 14439903

[23] Kleinknecht D, Rollin PE. Hypertension after hemorrhagic fever with renal syndrome. Nephron. 1992;61:121. 10.1159/000186853 1356239

[24] Krautkrämer E, Grouls S, Urban E, Schnitzler P, Zeier M. No gender-related differences in the severity of nephropathia epidemica, Germany. BMC Infect Dis. 2013;13:457. 10.1186/1471-2334-13-457 24090247 PMC3850742

[25] Pickering TG, Hall JE, Appel LJ, Falkner BE, Graves J, Hill MN, Recommendations for blood pressure measurement in humans and experimental animals: part 1: blood pressure measurement in humans: a statement for professionals from the Subcommittee of Professional and Public Education of the American Heart Association Council on High Blood Pressure Research. Hypertension. 2005;45:142–61. 10.1161/01.HYP.0000150859.47929.8e 15611362

[26] Chobanian AV, Bakris GL, Black HR, Cushman WC, Green LA, Izzo JL Jr, The seventh report of the Joint National Committee on Prevention, Detection, Evaluation, and Treatment of High Blood Pressure: the JNC 7 report. JAMA. 2003;289:2560–72 . 10.1001/jama.289.19.2560 12748199

[27] Bellomo R, Ronco C, Kellum JA, Mehta RL, Palevsky P. Acute renal failure—definition, outcome measures, animal models, fluid therapy and information technology needs: the Second International Consensus Conference of the Acute Dialysis Quality Initiative (ADQI) Group. Crit Care. 2004;8:R204–12. 10.1186/cc2872 15312219 PMC522841

[28] Outinen TK, Tervo L, Mäkelä S, Huttunen R, Mäenpää N, Huhtala H, Plasma levels of soluble urokinase-type plasminogen activator receptor associate with the clinical severity of acute Puumala hantavirus infection. PLoS ONE. 2013;8:e71335. 10.1371/journal.pone.0071335 23990945 PMC3749226

[29] Outinen TK, Mäkelä SM, Ala-Houhala IO, Huhtala HS, Hurme M, Paakkala AS, The severity of Puumala hantavirus induced nephropathia epidemica can be better evaluated using plasma interleukin-6 than C-reactive protein determinations. BMC Infect Dis. 2010;10:132. 10.1186/1471-2334-10-132 20500875 PMC2885391

[30] Krautkrämer E, Grouls S, Stein N, Reiser J, Zeier M. Pathogenic old world hantaviruses infect renal glomerular and tubular cells and induce disassembling of cell-to-cell contacts. J Virol. 2011;85:9811–23. 10.1128/JVI.00568-11 21775443 PMC3196447

[31] Vaheri A, Strandin T, Hepojoki J, Sironen T, Henttonen H, Mäkelä S, Uncovering the mysteries of hantavirus infections. Nat Rev Microbiol. 2013;11:539–50. 10.1038/nrmicro3066 24020072

[32] Turčinov D, Puljiz I, Markotić A, Kuzman I, Begovac J. Clinical and laboratory findings in patients with oliguric and non-oliguric hantavirus haemorrhagic fever with renal syndrome: an analysis of 128 patients. Clin Microbiol Infect. 2013;19:674–9. 10.1111/j.1469-0691.2012.03994.x 22963396

[33] Heyman P, Vaheri A, Lundkvist A, Avsic-Zupanc T. Hantavirus infections in Europe: from virus carriers to a major public-health problem. Expert Rev Anti Infect Ther. 2009;7:205–17. 10.1586/14787210.7.2.205 19254169

[34] Hautala N, Kauma H, Vapalahti O, Mahonen SM, Vainio O, Vaheri A, Prospective study on ocular findings in acute Puumala hantavirus infection in hospitalised patients. Br J Ophthalmol. 2011;95:559–62. 10.1136/bjo.2010.185413 20679079

[35] Wolf-Maier K, Cooper RS, Banegas JR, Giampaoli S, Hense HW, Joffres M, Hypertension prevalence and blood pressure levels in 6 European countries, Canada, and the United States. JAMA. 2003;289:2363–9. 10.1001/jama.289.18.2363 12746359

[36] Elgh F, Linderholm M, Wadell G, Tarnvik A, Juto P. Development of humoral cross-reactivity to the nucleocapsid protein of heterologous hantaviruses in nephropathia epidemica. FEMS Immunol Med Microbiol. 1998;22:309–15. 10.1111/j.1574-695X.1998.tb01220.x 9879922

[37] Elgh F, Lundkvist A, Alexeyev OA, Stenlund H, Avsic-Zupanc T, Hjelle B, Serological diagnosis of hantavirus infections by an enzyme-linked immunosorbent assay based on detection of immunoglobulin G and M responses to recombinant nucleocapsid proteins of five viral serotypes. J Clin Microbiol. 1997;35:1122–30 . 9114393 10.1128/jcm.35.5.1122-1130.1997 PMC232715

[38] Maes P, Clement J, Gavrilovskaya I, Van Ranst M. Hantaviruses: immunology, treatment, and prevention. Viral Immunol. 2004;17:481–97. 10.1089/vim.2004.17.481 15671746

[39] Klempa B, Meisel H, Rath S, Bartel J, Ulrich R, Kruger DH. Occurrence of renal and pulmonary syndrome in a region of northeast Germany where Tula hantavirus circulates. J Clin Microbiol. 2003;41:4894–7. 10.1128/JCM.41.10.4894-4897.2003 14532254 PMC254384

[40] Klempa B, Schutt M, Auste B, Labuda M, Ulrich R, Meisel H, First molecular identification of human Dobrava virus infection in central. J Clin Microbiol. 2004;42:1322–5. 10.1128/JCM.42.3.1322-1325.2004 15004109 PMC356881

