# Supplementary material for: Clinical Course and Long-Term Outcome of Hantavirus-Associated Nephropathia Epidemica, Germany
Source: Emerg Infect Dis. 2015 Jan;21(1):76–83. doi: 10.3201/eid2101.140861 (PMC4285283; doi:10.3201/eid2101.140861)
Supplement: Technical Appendix — Details of data acquisition from patients during acute course of nephropathia epidemica and flowchart showing design of hantavirus study, Germany, 2001–2012. [file 14-0861-Techapp-s1.pdf]

Article DOI: http://dx.doi.org/10.3201/eid2101.140861

Clinical Course and Long-Term Outcome of Hantavirus-Associated Nephropathia Epidemica, Germany, 2001–2012

Technical Appendix

Detailed Data Acquisition during Acute Course of the Disease

Clinical and laboratory data during the acute course of the disease were obtained from medical reports and files from each patient. At time of discharge from hospital, clinical signs and symptoms were recorded from medical charts and from the patients at follow-up. Information on oliguria, anuria, and polyuria in the course of the disease were obtained from the documented file information.

The following laboratory values were assessed from admission to discharge in hospitalized patients and at different time points in ambulatory patients.

Maximum values for serum creatinine, minimum values for platelets, maximum values for C-reactive Protein (CRP), lactate dehydrogenase (LDH), absolute maximum values for serum creatinine, CRP and, and absolute minimum values for platelets LDH when available. Absolute maximum and minimum values were defined if an increase or decrease to a peak level or nadir followed by a decline could be documented. In addition to the above, liver function tests, blood count, uric acid, procalcitonin and data for urine analyses were noted.

Detailed Data Acquisition at Time of Follow-Up

All patients (in-patients and ambulatory patients) were included in the follow-up. Patients were followed once at the outpatient department at the Robert-Bosch-Hospital, Stuttgart, Germany. Two readings of systolic blood pressure and diastolic blood pressure, separated by intervals of at least 5 minutes were measured in the supine position after at least 15 minutes of resting. The average of two readings of blood pressure was classified as normotensive for systolic blood pressure systolic (SBP) <120 and diastolic blood pressure (DBP) >80 mm Hg).

Prehypertension was classified by SBP 120–139 or DBP 80–89 mm Hg; hypertension stage 1 by SBP of 140–159 mm Hg or DBP 90–99 mm Hg; and hypertension stage 2 by SBP >160 mm Hg or DBP >100 mm Hg according to the classification of blood pressure for adults of the Seventh Report of the Joint National Committee on Prevention, Detection, Evaluation, and Treatment of High Blood Pressure. Physicians and study nurses reviewed charts for patients with stage 1 and 2 hypertension at time of follow-up and contacted these patients by telephone within 8 weeks after appointment in the outpatient clinic. In this interview, blood pressure measurements at home or at general practitioner and change in antihypertensive drug medication were noted. Using averages of these measurements, a reclassification of the patients with hypertension stage 1 and 2 was made.

History of pre-existing hypertension and medication were noted for all patients. In patients with pre-existing hypertension it was noted whether hypertension aggravated after hantavirus infection.

At the follow-up appointment, complete blood count (CBC), plasma CRP, serum creatinine, urea, liver function tests and blood gas analysis were measured at the Laboratory Centre of the Robert-Bosch-Hospital, Stuttgart, using standard methods. Second-morning urine specimens were analyzed for osmolality and were tested for erythrocytes, leukocytes, albumin, nitrite, glucose, pH, and ketones. Urine was analyzed for the presence of microalbumin. Spot urine protein-creatinine ratios (g/g) were determined. Proteinuria was defined as microalbuminuria (>30 mg albumin/24 h) in urine. Exclusion criteria for proteinuria were of bacteria in urine and menorrhea.

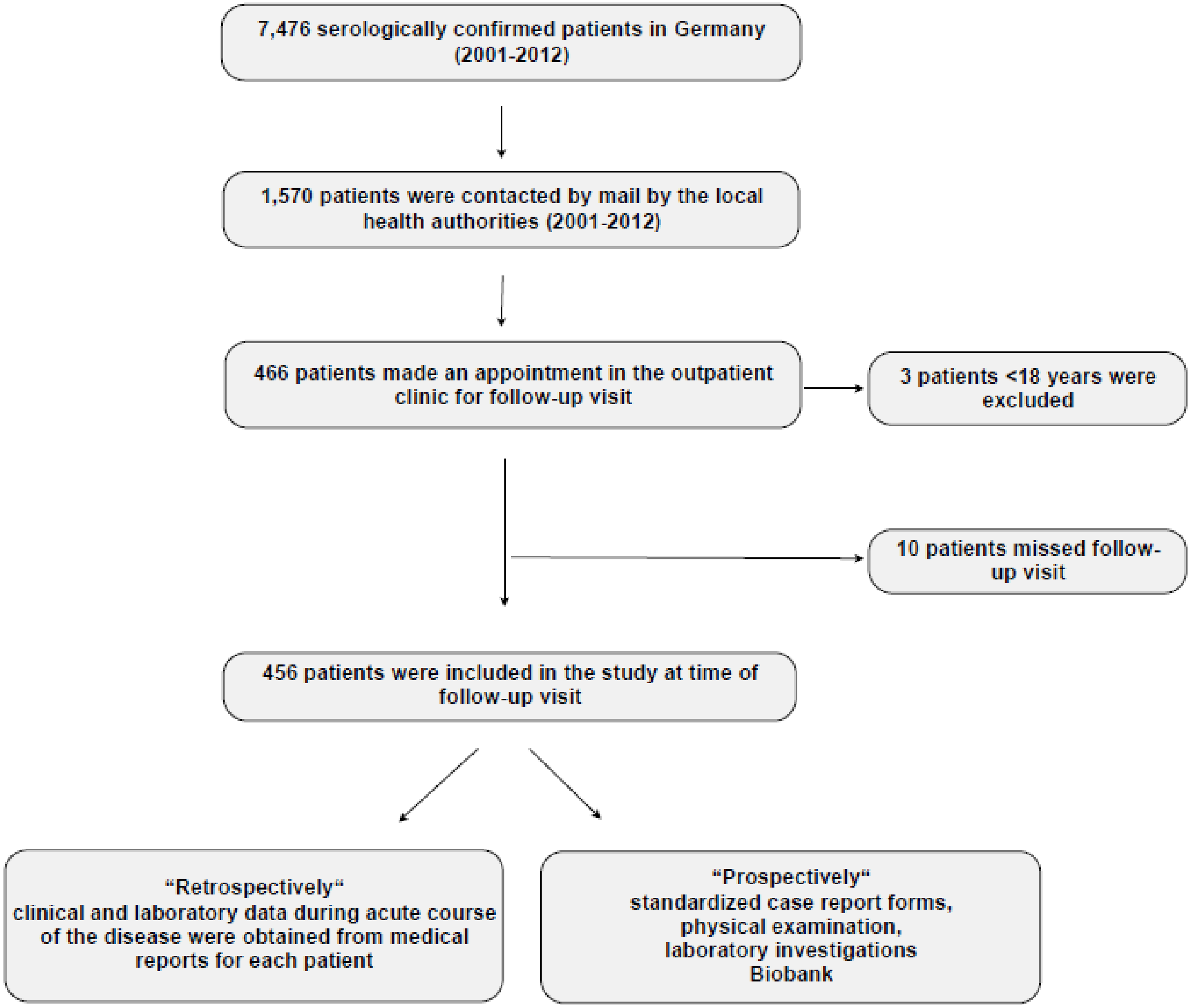

Technical Appendix Figure. Design of hantavirus study, Germany, 2001–2012. Biobank included additional blood samples and urine samples for further analyses.
